# PUFA Treatment Affects C2C12 Myocyte Differentiation, Myogenesis Related Genes and Energy Metabolism

**DOI:** 10.3390/genes12020192

**Published:** 2021-01-28

**Authors:** Marua Abu Risha, Puntita Siengdee, Dirk Dannenberger, Klaus Wimmers, Siriluck Ponsuksili

**Affiliations:** 1 Functional Genome Analysis Research Unit, Institute of Genome Biology, Leibniz Institute for Farm Animal Biology (FBN), Wilhelm-Stahl-Allee 2, D-18196 Dummerstorf, Germany; risha@fbn-dummerstorf.de (M.A.R.); siengdee@fbn-dummerstorf.de (P.S.); 2 Genomics Research Unit, Institute of Genome Biology, Leibniz Institute for Farm Animal Biology (FBN), Wilhelm-Stahl-Allee 2, D-18196 Dummerstorf, Germany; wimmers@fbn-dummerstorf.de; 3 Lipid Metabolism and Muscular Adaptation Workgroup, Institute of Muscle Biology and Growth, Leibniz Institute for Farm Animal Biology (FBN), Wilhelm-Stahl-Allee 2, D-18196 Dummerstorf, Germany; dannenberger@fbn-dummerstorf.de; 4 Faculty of Agricultural and Environmental Sciences, University Rostock, 18059 Rostock, Germany

**Keywords:** C2C12, energy metabolism, glycolysis, oxidative phosphorylation, PUFA, fatty acids, myogenesis, Wnt signaling

## Abstract

Polyunsaturated fatty acids (PUFAs) are the main components of cell membrane affecting its fluidity, signaling processes and play a vital role in muscle cell development. The effects of docosahexaenoic acid (DHA) on myogenesis are well known, while the effects of arachidonic acid (AA) are largely unclear. The purpose of this study is to evaluate the effect of two PUFAs (DHA and AA) on cell fate during myogenic processes, Wnt signaling and energy metabolism by using the C2C12 cells. The cells were treated with different concentrations of AA or DHA for 48 h during the differentiation period. PUFA treatment increased mRNA level of myogenic factor 5 (*Myf5*), which is involved in early stage of myoblast proliferation. Additionally, PUFA treatment prevented myoblast differentiation, indicated by decreased myotube fusion index and differentiation index in parallel with reduced mRNA levels of myogenin (*MyoG*). After PUFA withdrawal, some changes in cell morphology and myosin heavy chain mRNA levels were still observed. Expression of genes associated with Wnt signaling pathway, and energy metabolism changed in PUFA treatment in a dose and time dependent manner. Our data suggests that PUFAs affect the transition of C2C12 cells from proliferation to differentiation phase by prolonging proliferation and preventing differentiation.

## 1. Introduction

Myogenesis is a complex process involving many events including proliferation, differentiation and migration of myoblasts, and is regulated by several signaling pathways such as Wnt, BMP and Shh [1,2]. Myogenic regulatory factors (MRFs) are a part of this complex network and regulate embryonic myogenesis, drive muscle regeneration, and repair damage by activating satellite cells. MRFs are specific transcriptional activator factors of muscle-specific genes including myogenin (MyoG), myogenic myogenic differentiation (MyoD), myogenic factor 5 (Myf5) and myogenic factor 6 (Myf6) [3]. Myf5 is the earliest MRF to be expressed during embryonic development [4]. Myf5 enhances myoblasts expansion, while MyoD determines myoblast differentiation prospect. MyoD and MyoG acts together with myocyte enhancer factor 2 (MEF2) to drive differentiation [5]. Myf6 regulates the final differentiation of myotubes [6]. Besides this complex network, cell membrane properties (receptors, channels, lipid composition and polarity) are also considered strong players in myogenesis [7] and key elements for maintaining cell physiology and cell survival. Cell membranes consist of two layers which are primarily composed of phospholipids and also contain cholesterols and sphingolipids [8]. The diversity of membrane depends on the fatty acids in the tails of phospholipids. In particular, polyunsaturated fatty acids (PUFAs), long fatty acids with more than one double bond, can change the functionality of membranes. Both n-3-PUFAs (ω-3) and n-6-PUFAs (ω-6) are considered important molecular components regulating various functions such as anti-inflammatory and pro-inflammatory properties [9,10]. A recent study reported that arachidonic acid (AA, C20:4 n-6) and eicosapentaenoic acid (EPA, C20:5 n-3) reduce cholesterol efflux from cholesterol-loaded macrophages, while docosahexaenoic acid (DHA, C22:6 n-3) had no impact on the mouse macrophages [11]. Many fatty acids including oleic acid (OA), linoleic acid (LA), γ-linoleic acid (GLA), arachidonic acid (AA) and cis-9, trans-11 conjugated linoleic acid (c9, t11 CLA) can modulate myoblast proliferation and myogenic differentiation [12,13]. EPA and DHA pose an inhibitory effect on myoblast proliferation and differentiation, and downregulate muscle-related gene expression [14].

Fatty acids intake changes skeletal muscle lipid composition and membrane fluidity by incorporating into the membrane and remodeling the microdomains, which can affect many cellular signaling processes [15,16,17,18]. Wnt signaling pathway is crucial for myogenesis [19], and such signaling pathways are highly active during embryonic development [20] and muscle regeneration [21]. Wnt signaling pathway is not only involved in to cell proliferation, differentiation, migration and survival, but also regulates self-renewal of stem cells and tumorigenesis [22,23]. A previous study reported that some n-3 PUFAs reduce oxidative stress [24], possibly by suppressing lipid peroxidation [25]. Most studies focused on the biological effects of EPA and DHA on C2C12 myoblast proliferation and differentiation and the results are still inconsistent [26]. In addition, the link between myogenesis related gene including Wnt signaling and energy metabolism on C2C12 myoblast proliferation and differentiation after modulation with AA and DHA is still unclear. In this study, we used C2C12 cells as a model for the myogenesis to evaluate the effect of different concentrations of DHA and AA on myogenesis, regulation of Wnt signaling and energy metabolism.

## 2. Materials and Methods

### 2.1. Cell Culture

C2C12 cells passage 11 (ATCC^®^ CRL1772™, LGC Standards GmbH, Wesel, Germany) were maintained in complete growth medium (GM containing Dulbecco’s Modified Eagle’s medium [+] 4.5 g/L d-Glucose, l-Glutamine [−] Pyruvate (DMEM, Gibco, New York, NY, USA), supplemented with 10% fetal bovine serum (FBS, Sigma-Aldrich, Taufkirchen, Germany) and 1% Antibiotic Antimycotic solution (Sigma-Aldrich, Taufkirchen, Germany) at 37 °C under 5% CO_2_. The cells were detached using 0.125% Trypsin-EDTA (Biochrom, Berlin, Germany).

### 2.2. PUFA Preparation for In Vitro Culture

PUFAs used in our experiment were prepared by conjugating either docosahexaenoic acid (Sigma-Aldrich, D2534) or arachidonic acid (Sigma-Aldrich, A3611) with fatty acid free bovine serum albumin (BSA) at 4:1 ratio and stored at −20 °C. The fatty acids were mixed with 1 mL methanol and evaporated under nitrogen gas stream. BSA/PBS solution was added to the mix, sonicated for 1 hour, and pH was adjusted to 7.4 with 1 N sodium hydroxide (NaOH).

### 2.3. Assessment of PUFA Effect on Cell Viability by xCELLigence Real-Time Assay

The xCelligence Real Time Cell Analyzer (RTCA) System was used to determine cell viability in real-time as reflected by cell proliferation. The optimum PUFA concentration and duration of treatment were first tested by measuring the cell index. In this experiment, 5000 cells/well were seeded in 16-well plates with complete growth medium under above described conditions. The cells were allowed to attach for 5 h and then AA or DHA was added to the wells at four concentrations (20, 30, 50, 100 μM). Equivalent concentrations of BSA (4.7, 7.1, 11.8 and 23.7 μM) were used as positive controls. Three replicates were used for each treatment and cellular impedance was measured every 0.5 h for 72 h. The experimental code is described in Table 1. The data from xCelligence were collected with the system software. Normalized cell index is the cell index value adjusted to 1 at the start time of PUFA treatment. The normalization function of the software converts all values to a proportion of 1 [27]. The non-toxic concentration of PUFAs for C2C12 was determined based on their negative impact on cell proliferation.

### 2.4. Cell Differentiation Experiment

**Figure 1:**
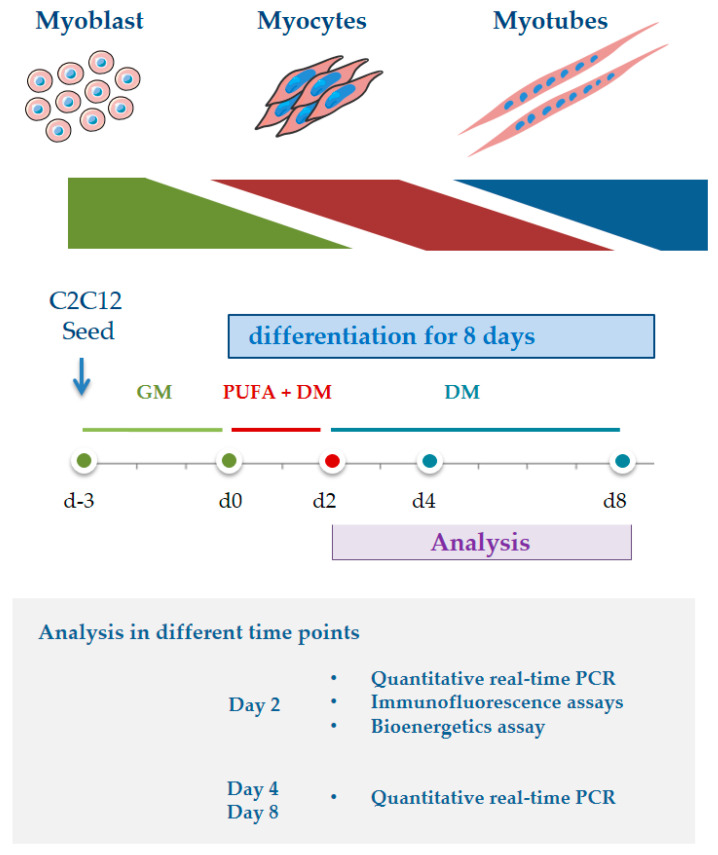
Summary of the experimental design of differentiation experiment. GM = growth medium; DM = differentiation medium.

The C2C12 of cell passage 11 were seeded in 6-well/plates in room temperature for 30 min to assure the attachment on the plastic surface, and then cultured at 37 °C under 5% CO_2_. To induce differentiation, culture medium was switched to differentiation medium (DM) Dulbecco’s Modified Eagle’s medium [+] 4.5 g/L d-Glucose, l-Glutamine [−] Pyruvate (DMEM, Gibco), supplemented with 2% horse serum (HS, Sigma) and 1% Antibiotic Antimycotic solution (Sigma) at 37 °C under 5% CO_2_. DHA and AA were added to DM in two concentrations (20 and 50 μM) separately. BSA was added in two concentrations correspond to the PUFA (4.74 μM (BSA20) and 11.85 μM (BSA50)). After 48 h of treatment, the culture medium was switched back to DM without PUFA. Cells were collected for RNA isolation at different time points; differentiated cells (d2) after 48 h of treatment, after 48 h treatment plus 2 days of recovery (d4), and after 48 h treatment plus 6 day of recovery (d8) (Figure 1). Three independent replicates were performed for each group.

### 2.5. Quantitative Real-Time PCR

Total RNA was isolated from each treatment group using TRI reagent (Sigma-Aldrich, Taufkirchen, Germany) according to the manufacturer’s instructions, followed by a purification step using RNeasy MiniKi (Qiagen, Hilden, Germany) and DNase I to remove any trace of DNA. Quantitative reverse transcriptase PCR (RT-qPCR) was performed using Superscript II reverse transcriptase (Invitrogen, Carlsbad, CA, USA), oligo(dT), with specific target amplification (STA) and Exonuclease I (Exo-I) treatment. The qPCR analyses were performed via BioMark HD Real-time PCR System (Fluidigm, South San Francisco, CA, USA) using 48 × 48 dynamic array with an integrated fluidic circuit (IFC). For the assay mix, 2.5 μL assay loading reagent, 2.3 μL DNA suspension buffer and 0.25 μL of a 100 μM primer solution were used (forward and reverse primers provided in Supplementary Table S1). The master mix for sample plate consists of 2.5 μL SsoFast EvaGreen supermix with low ROX (Biorad, Hercules, CA, USA), 0.25 μL DNA binding dye and 2.25 μL STA and Exo-I. The thermal conditions were 95 °C for 60 s for initial denaturation, 30 cycles of denaturation at 95 °C for 5 s and annealing at 60 °C for 20 s. Data analysis was done by the 2^−ΔCt^ method. The reference genes *Gapdh* and *Mrps27* were used as housekeeping control, which showed no significant changes in DHA, AA or BSA treatments. Selection of candidate Wnt pathway genes were based on their role in the pathway. It included inhibitors (*Wif1*, *Dkk1* and *Sfrp1*), regulators (*rspo1*, *Cby1*, *Tnks2*, *Porcn* and *Znrf3*), specific components of Wnt/PCP pathway (*Ror2*, *Daam1*, *FZD7* and *Rac1*), Wnt/Ca^+2^ pathway (*Ppp3ca*), Wnt/β-Catenin pathway (*Axin2*, *Lrp5*, *Lrp6* and *Csnk1g2*) and a key player in all Wnt pathways (*Dvl2*). Other genes were selected based on their role in metabolism (*PGC-1α*, *Prkaa1* and *Sirt1*) and myogenesis (*Myf5*, *Myf6*, *MyoD1*, *MyoG*, *Myf6*, *Myh1*, *Myh2* and *Myh4*). All genes and primers are listed in Supplementary Table S1. All experiments were performed three times independently.

### 2.6. Immunofluorescence Assays

After treatment with DHA, AA and BSA for 48 h, C2C12 cells were fixed in 4% formaldehyde for 10 min and then washed 3 times with PBS for 5 min each. The cells were permeabilized with 0.5% Triton X-100 for 10 min, followed with 3 washes with PBS for 5 min. Blocking was done for 1 h with blocking buffer consisting of 10% PBS, 1% BSA and 0.3% Triton X-100. Then, the cells were incubated with anti-myogenin Alexa488 green (1:300, 53-5643-82, eBioscience™ by Thermo Fisher, Schwerte, Germany) for one h at 4 °C in dark. After two PBS washes, cells were incubated with Hoechst 33342 (0.2 μg/mL, H1399, Invitrogen by Thermo fisher) for 5 min in dark. The cells were then washed once with PBS and once with water. Fluorescence imaging was obtained with CC-12 high-resolution color camera (OSIS) connected to Nikon Microphot SA fluorescence microscope (Nikon) and analyzed with CELL^F software. For each treatment multiple photos were taken and in each field. Myotubes number, number of nuclei incorporated in myotubes, and the total number of nuclei were scored. The differentiation index was calculated by counting the number of nuclei showing Alexa 488 staining divided by the total number of nuclei from the same field. The Fusion index was calculated by counting the number of nuclei in myotubes containing more than 2 nuclei divided by the total number of nuclei from the same field.

### 2.7. Bioenergetics Assay

C2C12 cells were incubated with DHA, AA or BSA for 48 h to differentiate to myotubes. For measuring energy metabolism, the treated cells were processed to measure glycolytic activity/extracellular acidification rate (ECAR) with the Seahorse XF Glycolysis kit, and mitochondrial function/oxygen consumption rate (OCR) with the Seahorse Cell Mito Stress Test kit. Glycolysis Stress Test is the standard assay for measuring glycolytic function in cells via the direct measurement of the ECAR in real time. Therefore glucose, oligomycin, and 2-deoxyglucose were added sequentially to reveal and analyses the key function of the glycolytic pathway. The XF Cell Mito Stress Test measures key parameters of mitochondrial function by directly measuring the oxygen consumption rate of cells. This test uses modulators of respiration targeting components of the electron transport chain in the mitochondria to reveal key parameters of metabolic function. After 2 days of treatments (BSA20, AA20, DHA20, BSA50, AA50 and DHA50), the growth medium including treatments were removed from the wells and replaced with XF Assay Medium. The details of this procedure were recently described by Sajjanar [28]. Bradford protein assay was used to measure protein in each well for normalization of OCR and ECAR values.

### 2.8. Data Analysis

The normalized data from xCELLigence, bioenergetics assay, differentiation index and fusion index were used as input for analysis using SAS programs. Treatment was considered as fixed effect. An adjustment for multiple comparisons across the Type 3 tests for the fixed effect was calculated using the post hoc Tukey–Kramer test and *p* < 0.05 was considered statistically significant. The gene expression levels were subjected to a mixed-model analysis of variance using JMP Genomics (Proc Mixed; SAS Institute, Rockville, MD, USA). Treatment and day were used as fixed effects. Additionally, replicates were used as random effect, *p* < 0.05 was considered statistically significant.

## 3. Results

### 3.1. Assessment of PUFA Effect on Cell Viability

**Figure 2:**
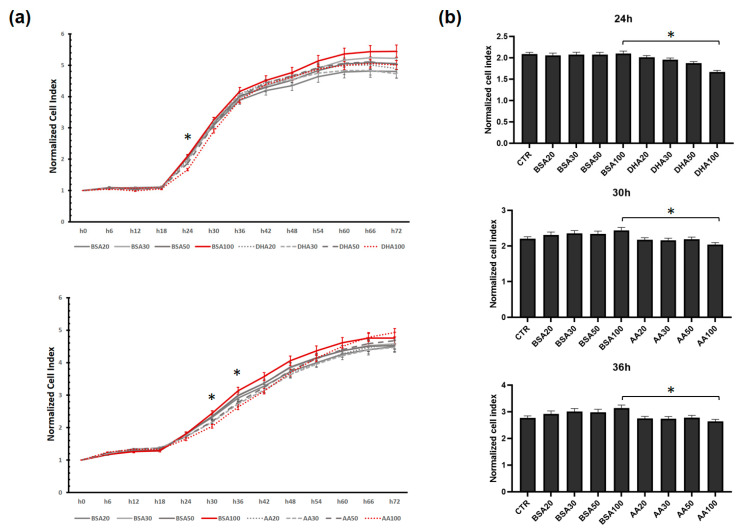
The effect of PUFA treatment on C2C12 cell proliferation. The changes were observed in real-time with the xCELLigence system after DHA and AA treatments. (**a**) The normalized cell index for all treatments is shown with dot lines (DHA or AA) and the respective BSA control for each group with solid lines. The cells were exposed to 20, 30, 50 and 100 μM of DHA or AA. The red solid line represents the concentration of BSA100 and the red dot line represents the concentration of DHA100 or AA100. (**b**) Monitoring living cells showed three time points when PUFA treatments at high doses reduced C2C12 proliferation. After 24 h, the cell index showed a decrease with DHA100 (100 μM) but no changes in DHA20, DHA30 or DHA50. After 30 and 36 h, the cell index showed a significant decrease with AA100 but no changes were observed in AA20, AA30 or AA50 treatments. Error bars indicate standard error of the mean (SEM), the asterisk * indicate significance between PUFA and equivalent BSA control at *p <* 0.05. The scale of the Y-axis corresponds to the normalized cell index of (**a**) with the same time point. CTR refers to control, i.e., GM without AA or DHA or BSA.

The xCelligence RTCA system records cell impedance as a function of cell density and was used to monitor cell viability of C2C12 cells treated with PUFA (DHA or AA). Cell viability correlates with cell proliferation. Between 24 and 36 h the cells were in the growth phase and during this time the effect of the high PUFA concentration (100 μM) on cell proliferation was visible and significantly different from the control (Figure 2a). When compared with control (BSA), the cell proliferation, shown as cell index, was significant lower with the addition of DHA (100 μM) after 24 h, while the same effect was monitored on 100 μM AA after 30 h and 36 h (Figure 2b). Other concentrations of PUFAs (20, 30 and 50 μM) did not affect cell proliferation compared to control (BSA) during 3 days of treatment. Based on these results, we decided to use PUFAs at 20 and 50 μM concentrations for further experiments.

### 3.2. AA and DHA Reduced Myogenic Differentiation

**Figure 3:**
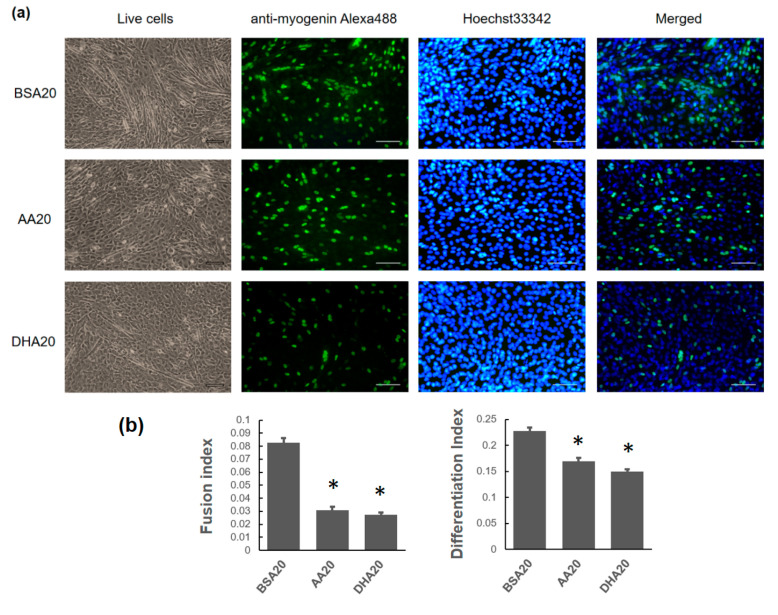

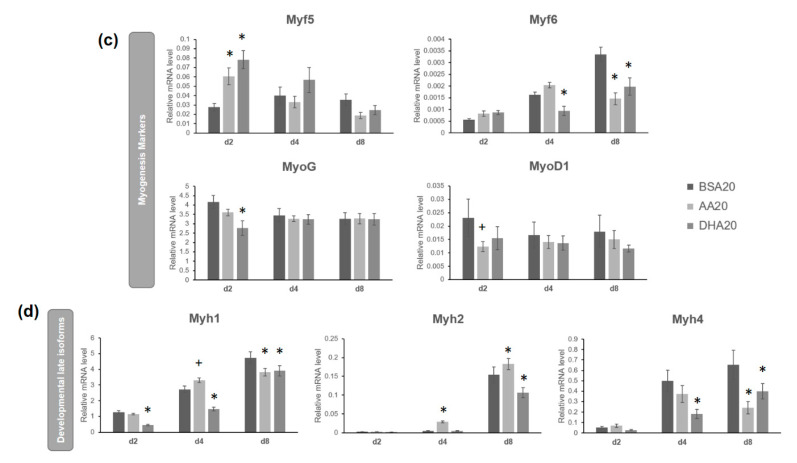
The effect of 20 μM PUFA on C2C12 myotubes formation. (**a**) Immunofluorescence staining using Anti-Myogenin Alexa Fluor^®^ 488 and Hoechst 33342 staining on C2C12 myotubes at day 2 (d2) of the treatment. Scale bar for live cells is 100 μm, Scale bar for anti-myogenin Alexa488, Hoechst 33342 staining and merged is 200μM. (**b**) Quantification of differentiation index and fusion index are shown. The mRNA level of myogenesis markers at three time points (d2 (2 days treatment), d4 (2 days treatment plus 2 days recovery), d8 (2 days treatment plus 6 days recovery)) was showed. (**c**) At d2, *Myf5* expression level was induced in both AA20 and DHA20 treatments. *MyoG* expression level was significantly decreased in DHA20 treatment. *MyoD1* expression level was decreased in AA20 treatment. The expression of *Myf6* significantly decreased at d4 in DHA20, and significantly decreased in both AA20 and DHA20 at d8. (**d**) The expression levels of *Myh1* significantly decreased at d2, d4 and d8 in DHA20 and in AA20 at d8. The expression levels of *Myh2* significantly increased in d4 and d8 in AA20 and significantly decreased in DHA20 at d8. The expression levels of *Myh4* significantly decreased at d4 in DHA20 and in both AA20 and DHA20 at d8. All values are LS means ± SEM, + indicate *p* < 0.1 and * indicates *p* < 0.05 treatment vs. control group BSA20.

**Figure 4:**
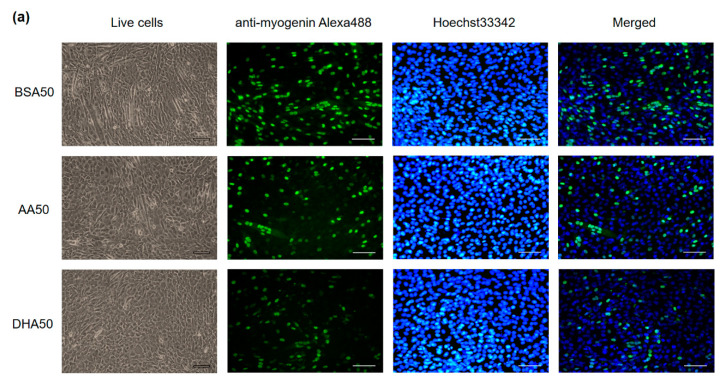

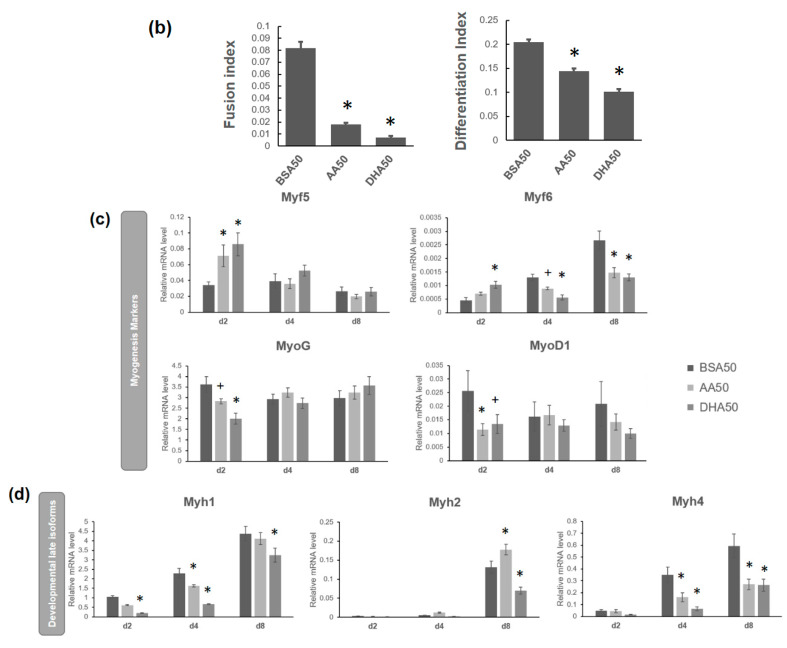
The effect of 50 μM PUFA on C2C12 myotubes formation. (**a**) Immunofluorescence staining using Anti-Myogenin Alexa Fluor^®^ 488 and Hoechst 33342 staining on C2C12 myotubes at day 2 (d2) of the treatment. Scale bar for live cells is 100 μm, Scale bar for anti-myogenin Alexa488, Hoechst 33342 staining and merged is 200 μM. (**b**) Quantification of differentiation index and fusion index are shown. The mRNA level of myogenesis markers at three time points (d2 (2 days treatment), d4 (2 days treatment plus 2 days recovery), d8 (2 days treatment plus 6 days recovery)) was shown. (**c**) At d2, *Myf5* expression level was induced in both AA50 and DHA50 treatment. *MyoG* expression level was significantly decreased in both AA50 and DHA50 treatment. *MyoD1* expression level was significantly decreased in AA50 and DHA50 treatments. The expression level of *Myf6* was significantly increased in DHA50 at d2 and decreased at d4, but significantly decreased in both AA50 and DHA50 at d8. (**d**) The expression level of *Myh1* was significantly decreased in d2, d4 and d8 in DHA50, also significantly decreased in AA50 at d4. The expression level of *Myh2* was significantly increased in AA50 and significantly decreased in DHA50 at d8. The expression levels of *Myh4* significantly decreased at d4 and d8 in both AA50 and DHA50. All values are LS means ± SEM, + indicate *p* < 0.1 and * indicates *p* < 0.05 treatment vs. control group BSA50.

After 2 days of treatments, all concentrations of AA or DHA (AA20, AA50, DHA20 and DHA50) led to a significant reduction in myogenic differentiation of C2C12 cells compared to control (Figure 3a,b or Figure 4a,b). The differentiated cells nuclei showed bright green color of anti-myogenin Alexa488 staining. The differentiation efficiency of AA20, AA50, DHA20 and DHA50 treated cells was reduced to 25%, 29%, 34% and 50% respectively compared to control. The fusion efficiency of AA20, AA50, DHA20 and DHA50 treated cells was reduced to 62%, 77%, 66% and 90%, respectively, compared to control. In addition, the results showed that DHA treatment causes higher reduction in myogenic differentiation compared to AA treatment. 

The level of *Myf5* mRNA was significantly increased whereas *MyoG* mRNA was significantly decreased at 2 days of AA20, DHA20, AA50 and DHA50 treatment (Figure 3c or Figure 4c). The transcript level of *Myf6* was significantly increased in DHA50 and of *Myh1* was significantly decreased in DHA20 and DHA50 after 2 days of treatment (Figure 3d or Figure 4d). The transcript level of *MyoD1* tended to be reduced compared to control (BSA) and was significantly reduced only at d2 with AA50 and DHA50 treatment. After 4 days of treatment, the changes in transcript abundances were significant for *Myf6* (DHA20, AA50, DHA50), *Myh1* (DHA20, AA50, DHA50), *Myh2* (AA20, DHA20, AA50, DHA50), and *Myh4* (DHA20, AA50, DHA50). Until six days after removal of PUFA treatment (d8), AA and DHA still affected myogenesis and *Myf6* mRNA was decreased in all treatments (Figure 3c or Figure 4c). In addition, changes in mRNA level of myosin heavy chain markers *Myh1*, *Myh2* and *Myh4*, which are more specific to fiber types, were obvious in all treatments except for *Myh1* in AA50.

### 3.3. Morphological Changes after 6 Days Recovery

**Figure 5:**
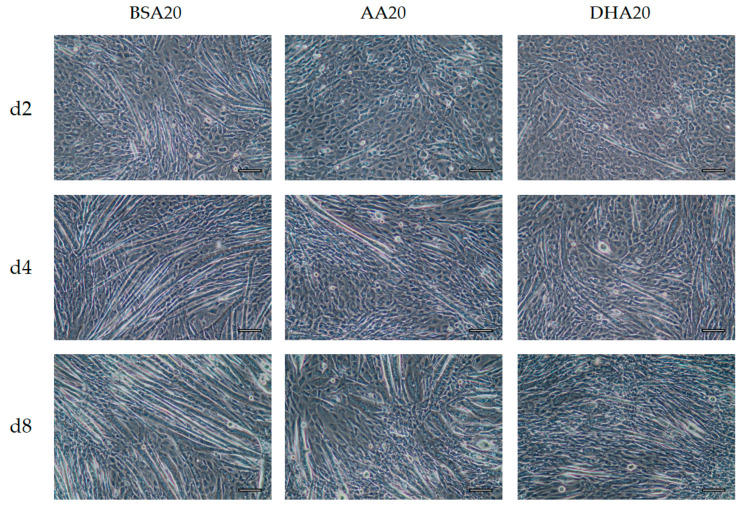
The phase contrast images of C2C12 differentiation stages under different treatments at different time points (Day 2 (d2), Day 4 (d4) and Day 8 (d8)). BSA20, AA20 and DHA20 treatments were added only on the first 2 days and then switched with normal DM medium till day 8. Scale bar 100 μm.

**Figure 6:**
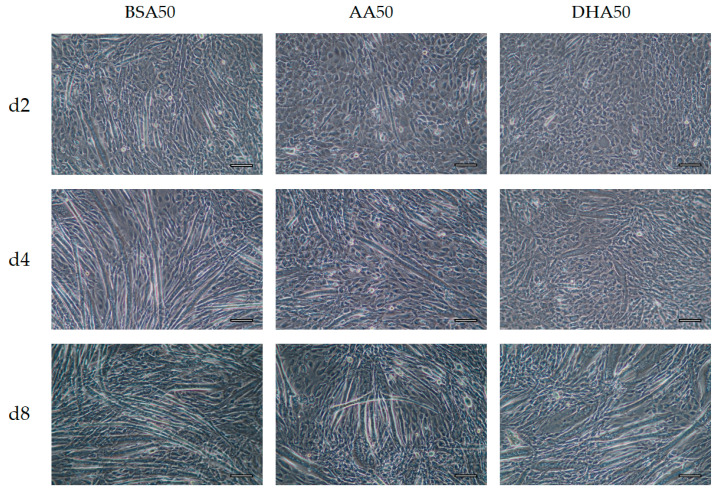
The phase contrast images of C2C12 differentiation stages under different treatments at different time points (Day 2 (d2), Day 4 (d4) and Day 8 (d8)). BSA50, AA50 and DHA50 treatments were added only on the first 2 days and then switched with normal DM medium till day 8. Scale bar 100 μm.

Our data show that AA and DHA treatment changed the myogenic differentiation (Figure 5 and Figure 6). Both AA20 and DHA20 slowed differentiation after 2 days of treatment. However, after switching back to only DM, the treated cells showed low numbers of myotubes formation on d4 than on d8. This was confirmed by transcript levels of developmental late isoforms (*Myh1*, *Myh2* and *Myh4*) which were lower on d4 and higher on day 8. In BSA control, more elongated myotubes were observed compared to treated cells. These results show that AA and DHA treatments induced changes in the cell morphology and caused a delay in the differentiation timeline of cells.

### 3.4. AA and DHA Affects the mRNA Abundance of Genes Involved in Wnt Signaling Pathway

**Figure 7:**
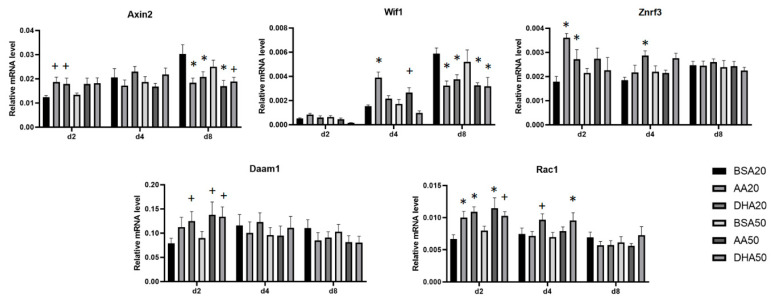
The mRNA levels of negative regulators of Wnt signaling pathway at three time points, i.e., 2 days treatment (d2), day 4 (d4; 2 days treatment + 2 days recovery) and day 8 (d8; 2 days treatment + 6 days recovery). The mRNA levels of *Axin* and *Znrf3* were increased at d2 relatively on all treated cells. The mRNA levels of *Wif1* and *Axin2* were decreased in all treated cells at day 8. All values are LS means ± SEM, + indicate *p* < 0.1 and * indicate *p* < 0.05 treatment vs. respective BSA control group.

Wnt signaling pathways are involved in cell differentiation. Selection of candidate Wnt pathway genes were based on their role in the pathway including inhibitors, regulators, receptor and key transcripts in specific parts of Wnt/PCP, Wnt/Ca+2, and Wnt/β-Catenin pathways. PUFA treated cells showed changes in mRNA level of three genes (*Axin2*, *Wif1* and *Znrf3*) involved in negative regulation of Wnt signaling pathway (Figure 7). *Axin2* mRNA was significantly increased after 2 days of AA20 and DHA20 treatments, however this increase did not reach significance in AA50 and DHA50 treatments. At day 8, *Axin2* mRNA level was significantly decreased in all treated cells (AA20, DHA20, AA50 and DHA50). There was a significant increase in *Wif1* mRNA in AA-treated samples at day 4, but it was significantly decreased at day 8. We found that, at day 2, the transcripts abundance of *Znrf3* was significantly increased in both AA20 and DHA20. Genes involved in Wnt/PCP pathway (*Daam1* and *Rac1)* were significantly increased at day 2.

### 3.5. AA and DHA Affects Myoblasts Energy Metabolism

**Figure 8:**
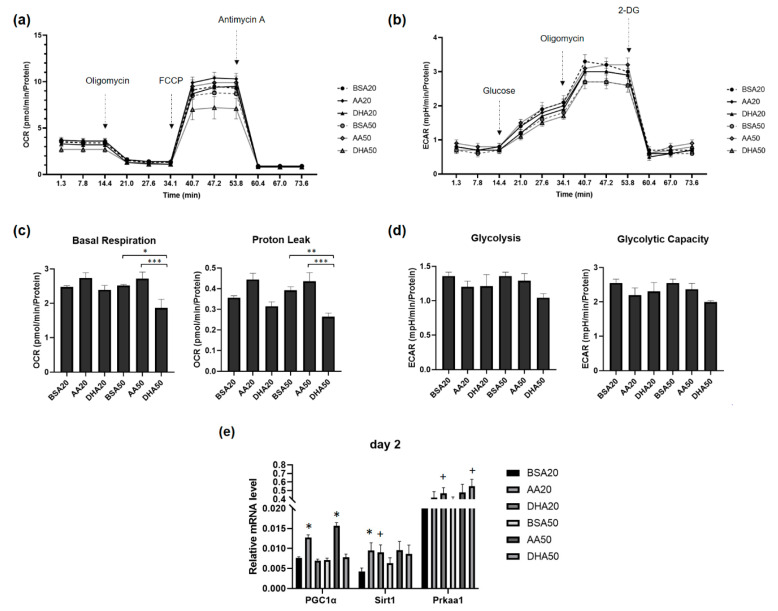
Bioenergetics metabolism in C2C12 under 2 days of AA and DHA treatments. (**a**) Mitochondrial respiration and (**b**) Glycolytic function in C2C12 cells in culture with different treatments; AA20, AA50, DHA20, DHA50, the controls BSA20 and BSA50. (**c**) The effect of AA50 and DHA 50 on the basal respiration and proton leak. (**d**) No effect of PUFA treatments on the glycolysis and glycolytic reserve. All data are from six well of 3000 cells/well per treatment. All values are LS means ± SEM given in pmol O_2_/min/μg protein. * p < 0.1, ** p < 0.05 and *** p < 0.001. (**e**) The mRNA levels of *PGC-1alpha*, *Sirt1* and *Prkaa1*; the mRNA levels of *PGC-1alpha* were significantly increased with AA treatments in both concentration (20 and 50 μM). The mRNA levels of *Prkaa1* were significantly increased with DHA20 and DHA50. Sirt1 mRNA levels were significantly increased with DHA and AA with 20 μM. All values are LS means ± SEM, + *p* < 0.1 and * *p* < 0.05.

To investigate whether DHA and AA treatments are involved in mitochondrial bioenergetics properties of C2C12 cells, the metabolic flux was analyzed by measuring the intracellular oxygen consumption rate (OCR). The OCR profiles before and after oligomycin, FCCP and antimycin-A injections in control (BSA) and treated cells (AA and DHA) were presented in Figure 8a. The ECAR profiles before and after glucose, oligomycin and 2-DG injections in control (BSA) and treated cells (AA and DHA) were shown in Figure 8b. No significant changes were found on the levels of basal respiration and proton leak of myoblasts after 48 h of treatment with AA20, DHA20 and AA50. However, both basal respiration and proton leak parameters were significantly lowered after treatment with DHA50 (Figure 8c). In addition, significant differences were found between AA and DHA treatments where AA seemed to increase OCR, while DHA tended to lower it in many mitochondrial parameters like basal respiration, ATP production and proton leak. There were no significant changes in glycolysis or glycolytic capacity of cells after PUFA treatments (Figure 8d). To explore the effect of AA and DHA on gene expression involved in mitochondrial biogenesis, we measured mRNA levels of *PGC-1alpha*, *Sirt1* and *Prkaa1* (Figure 8e). Significant changes in RNA expression were noticed in day 2; the transcripts level of *PGC-1alpha* was significantly increased in treated cells (AA20, AA50) compare to control (BSA20, BSA50) while *Sirt1* mRNA was significantly increased in AA20 and DHA20 compared to BSA20. The expression of *Prkaa1* was significantly increased in treated cells (DHA20 and DHA50) compared to control (BSA20, BSA50).

## 4. Discussion

N-3 and n-6 polyunsaturated fatty acids play an essential role in the development of skeletal muscle [29,30]. This study aimed to access the impact of PUFA on myogenesis and in particular on the major myogenic regulatory factors including *MyoG*, *MyoD1*, *Myf5* and *Myf6.* Previous studies have demonstrated both positive and negative impacts of PUFA on myogenesis. The positive influence of AA on the size of muscle cells in the existing myotubes has already been mentioned [31], and as well as the effect influence of other types of PUFAs on cell proliferation and differentiation has been previously described [12,13]. Previous studies have shown the role of n3-PUFA on muscle growth, regeneration, satellite cells programming, mitochondrial biosynthesis and modulation of skeletal muscle lipid raft composition [26,32,33,34]. The impact of a PUFA depends on the concentration used, duration of treatment, time point of treatment and the method of treatment [35]. In our experiment, we used BSA as a transporter for PUFA with a ratio of 1:4. Satellite cells are skeletal muscle cell precursors that can regenerate muscle cells during postnatal growth or regenerate skeletal muscle in adults in response to muscle injury. Muscle regeneration processes are sensitive and can be influenced by inflammatory processes [36]. Many n-3 PUFAs are known for their anti-inflammatory properties [37]. Evidence including 3n-PUFA’s role in satellite cells activation suggests the possible involvement of 3n-PUFA in the regeneration process during injury [26]. However, PUFA’s affects the transition period from proliferating phase to differentiation phase of muscle cells remains unclear. In this study, AA and DHA treatment affected *Myf5*, *MyoG*, *Myf6*, and *MyoD1*. *Myf5* mRNA was increased whereas *MyoG* and *MyoD1* mRNAs were decreased. *Myf5* facilitates early stage of myoblast proliferation [38]. As shown in our results, *Myf5* mRNA was reduced at day 4 and day 8. At day 2, the transcript level of *Myf5* was higher in the AA and DHA treated cells indicating increased myoblast proliferation. Overexpression of *Myf5* leads to upregulation of Cyclin D1, which reflects the transition from G1 to S stage in cell cycle [38,39]. DHA was reported to decrease mRNA and protein levels of both cyclin E and CDK2 during C2C12 proliferation and keeping the cells in G1 phase for longer [40]. MyoD and MyoG act together with myocyte enhancer factor 2 (MEF2) to drive myoblast differentiation [5]. MyoG expression marks the end of myoblast proliferation and induces differentiation of myoblasts including fusion to form multinucleated muscle myotubes [41,42]. Our data show that under PUFA treatment, there were fewer differentiated myotubes but more proliferative myoblasts as a result of prolonged proliferation and delayed differentiation. In contrast, in a study with L6C5 cells AA and DHA and, to a lesser extent, also EPA induced a significantly increased formation of myotubes after 4 days of incubation with 20 µM of PUFAs compared to the control cells [34]. However, it was not clear whether in this study the L6C5 myoblasts were differentiated before treatment. In another study, C2C12 myoblasts were subjected to 72 h of differentiation prior to AA supplementation, resulting in an increase in myotube size and protein content, but no differences in fusion and differentiation [31]. Recently, in a study with conditions similar to our study (48 h treatment with 25 or 50 μM DHA or EPA), a reduction in transcripts and proteins of myogenic marker was found in C2C12 myoblasts, suggesting that EPA and DHA suppress differentiation of C2C12 myoblasts [14]. Together with the state of the cell, the effect of PUFA treatment varies depending on the dose and time. 

Low concentration of DHA (10 μM) stimulates cell proliferation whereas higher concentration of DHA (100 μM) inhibits C2C12 cells proliferation [12,14]. In our study, real time monitoring of cell proliferation revealed that 100 μM DHA significantly reduced myoblasts proliferation particularly in the growth phase during 24 to 36 h of treatment, whereas no change was observed after PUFA treatment at 20, 30 and 50 μM doses during 3 days of treatment. In the cell differentiation experiment, mRNA level of *Myf5* was expressed higher at day 2 under PUFA treatment than in the control group. *Myf5* is involved in early phase of myoblast proliferation, suggesting the prolongation of proliferation phase under PUFA treatment. In addition, PUFA treatment prevented myoblast differentiation as showed by decreased myotube fusion index and myotube differentiation index paralleled with decrease in mRNA levels of *MyoG*. These data suggest the role of PUFAs in the transition period between proliferation and differentiation stages in myogenesis process.

Skeletal muscles consist of various fiber types making them heterogeneous with each fiber having its own properties [43]. In this study, three different markers from fast twitch fibers (*Myh1*, *Myh2* and *Myh4*) were chosen, that represent MyhHCIId/x, MyhHCIIa and MyhHCIIb fibers [44]. The transcripts levels of these genes increased along the culture days (d4 and d8) in the control groups and were overall reduced by AA and DHA even after two and four days of recovery. This is in line with reduced myotube number and fusion index after AA and DHA treatments at both concentrations (20 and 50 μM) along with a downregulation of *MyoG* mRNA expression level. This may play a substantial role in the expression of myosin heavy chain genes. The downregulation of Myf6 in late stage of the differentiation supports this claim. Our results are consistent with the previous study that showed the inhibitory effect of n-3 PUFAs on C2C12 myoblasts [14].

The potential effects of PUFAs on myogenesis may relate to their ability to alter the lipid composition of the cell membrane, and this alteration may lead to the modulation of various signaling pathways including Wnt signaling pathways. Wnt signaling pathways are key pathways in many biological processes including embryonic myogenesis [45,46]. Previous studies also showed the involvement of Wnt signaling cascades in development and maturation of different fiber types [44]. Therefore, we selected candidate genes based on their role in the Wnt pathway to study their response to PUFA treatment. Most of the transcripts in these pathways did not significantly change under PUFA treatment except *Daam1*, *Rac1* as elements of the non-canonical PCP Wnt signaling cascade at day 2 and *Axin2*, *Znrf3* and *Wif1, which* are involved in the Wnt/β-Catenin pathway. The high expression of *Daam1* and *Rac1* could impact cytoskeleton regulation [47] and also promote cell proliferation [48]. DAAM1 plays a critical role in regulating the actin cytoskeleton and tissue morphogenesis as shown in Daam1-deficient mice [49]. Wnt signaling is known to promote cell proliferation. In our study, the high expression of *Daam1* and *Rac1* under PUFA treatment may promote cell proliferation. Both AA and DHA treatments increased the expression of the negative regulators Wnt signaling (*Axin2* and *Znrf3)*. A previous study reported Axin2-dependent Wnt/β-catenin signaling is involved in myotube formation and associated with reduced muscle fiber diameter of a subset of fast fibers [46]. It has been previously reported that ω-3 PUFA negatively regulates nuclear and cytoplasmic level of β-Catenin in HepG2 cells [50]. In our study, AA particularly increased Wnt ligand inhibitor *Wif1*. WIF1 is considered a tumor suppressor and high concentration of n-3 PUFAs (above 100 µM) significantly increases *Wif1* expression in pancreatic cancer cell line (MIA PaCa-2) [51]. 

The proportion of cellular ATP production by glycolysis in the cytoplasm or oxidative phosphorylation (OXPHOS) in the mitochondria depends on many factors including stress condition [28]. In this study PUFA, treatment at doses of 20 or 50 µM has no effect on proliferation but prevented the myoblast differentiation. It is therefore interesting to know whether the mitochondrial bioenergetics properties of the mitochondria change under PUFA treatment. During stress response, the lactate-to-pyruvate ratios were increased, indicating a higher rate of glycolysis and reduced OXPHOS [52]. No significant change of the basal respiration, proton leak, glycolysis and glycolytic capacity was found under low concentration (20 µM) of both AA and DHA. The mitochondrial respiration slightly changed in the higher concentration (50 µM) of both AA and DHA. DHA treatments exhibit reduction in the basal respiration and proton leak in compared to the control BSA and both parameters were higher in AA treatment. DHA can incorporation into mitochondrial membranes [53]. These results suggest that high levels of DHA preserve mitochondrial integrity by incorporation into mitochondrial membranes.

PGC-1α is considered the master regulator for mitochondrial biogenesis [54]. The level of *PGC-1α* mRNA is significantly correlated with the expression of both mitochondrial and nuclear encoded OXPHOS subunits, as well as with the enzyme activities of complex I, II, and IV [55]. Interestingly, we found an increase in *Pgc-1α* mRNA levels in AA treated samples while expression levels of *Sirt1* was increased under both AA and DHA treatments. The tendency of intracellular oxygen consumption rate was also higher in AA treated samples. In a previous study, it was shown that the 20-carbon EPA (20:5n-3) and AA prompted higher incorporation into the mitochondrial inner membranes than 22-carbon DHA [56]. Fatty acid oxidation occurs within mitochondria, the higher expression of *PGC-1α* under AA treatment may be involve in this process. SIRT1 and AMPKα1 (*Prkaa1*) are nutrient sensors and their expressions and activities were reported to be increased after DHA treatment in macrophages [57]. Similar findings were reported in female Wistar rats fed with low proportion of n3 PUFA, where it showed an increase in *Sirt1* and *Pgc-1α* gene expression [58]. We found an increased in *Sirt1* mRNA levels in AA and DHA treated samples, which are consistent with previous studies. Two potential effects of PUFAs on energy metabolism may be related to their ability to incorporate into the inner mitochondrial membranes, and this change may lead to alteration in metabolic pathways in mitochondria.

In summary, our results show that PUFA treatment immediately increased mRNA levels of *Myf5*, which is involved in the early stage of myoblast proliferation. At the same time PUFA treatment decreased mRNA levels of *MyoG*. This is accompanied by prevented myoblast differentiation, as demonstrated by a decreased myotube fusion index and myotube differentiation index. Thus, this study of polyunsaturated fatty acids on myogenesis suggests that their influence is exerted on the disruption of the early phases. Some of the transcripts in the Wnt/β catenin and Wnt/PCP signaling pathways, including *Axin2, Znrf3, Wif1*, *Daam1* and *Rac1*, changed under PUFA treatment. No change in glycolysis and glycolytic capacity was observed under PUFA treatment. In addition, high DHA treatments showed a reduction in the basal respiration and proton leakage compared to the BSA control group, suggesting that high DHA concentrations may preserve mitochondrial integrity by incorporation into mitochondrial membranes. Finally, PUFA treatment increased key transcripts of mitochondrial energy metabolism including *Sirt1* and *PGC-1α* and *Prkaa1*.

## Figures and Tables

**Table 1:** The experimental code used in xCELLigence RTCA system.

Code	Treatment (Conditions)
AA20	Arachidonic acid 20 μM + BSA 4.7 μM
AA30	Arachidonic acid 30 μM + BSA 7.1 μM
AA50	Arachidonic acid 50 μM + BSA 11.8 μM
AA100	Arachidonic acid 100 μM + BSA 23.7 μM
DHA20	Docosahexaenoic acid 20 μM + BSA 4.7 μM
DHA30	Docosahexaenoic acid 30 μM + BSA 7.1 μM
DHA50	Docosahexaenoic acid 50 μM + BSA 11.8 μM
DHA100	Docosahexaenoic acid 100 μM + BSA 23.7 μM
BSA20	Bovine Serum Albumin 4.7 μM
BSA30	Bovine Serum Albumin 7.1 μM
BSAA50	Bovine Serum Albumin 11.8 μM
BSA100	Bovine Serum Albumin 23.7 μM

## Data Availability

The data (figures and table) used to support the findings of this study are included within the article.

## Supplementary Materials

The following are available online at https://www.mdpi.com/2073-4425/12/2/192/s1, Supplementary Table S1: Primers sequences used for amplification in qPCR. The table include information of primer sequence and the gene symbol.

## Abbreviations

PUFAs	Polyunsaturated fatty acids
DHA	Docosahexaenoic acid
AA	Arachidonic acid
Myh1	Myosin Heavy Chain 1
Myh2	Myosin Heavy Chain 2
Myh4	Myosin Heavy Chain 4
Myf6	Myogenic Factor 6
Myf5	Myogenic Factor 5
MyoG	Myogenin
MyoD	Myogenic myogenic differentiation
Daam1	Dishevelled Associated Activator of Morphogenesis 1
Znrf3	Zinc and Ring Finger 3
Wif1	Wnt Inhibitory Factor 1
Rac1	Rac Family Small GTPase 1
PGC-1α	PPARG Coactivator 1 Alpha
Prkaa1	Protein Kinase AMP-Activated Catalytic Subunit Alpha 1
Sirt1	Sirtuin 1
